# Families served during the first decade of the Supportive Services for Veteran Families program: a descriptive analysis

**DOI:** 10.3389/fpubh.2025.1634259

**Published:** 2025-07-24

**Authors:** Renae Wilkinson, Katelyn N. G. Long, Richard G. Cowden, Howard K. Koh, Thomas H. Byrne, Adrienne N. Melendez, Jack Tsai

**Affiliations:** ^1^Human Flourishing Program, Institute for Quantitative Social Science, Harvard University, Cambridge, MA, United States; ^2^Harvard T. H. Chan School of Public Health, Boston, MA, United States; ^3^Boston University School of Social Work, Boston, MA, United States; ^4^VA Edith Nourse Rogers Memorial Veterans Hospital, Bedford, MA, United States; ^5^US Department of Veterans Affairs Homeless Programs Office, Washington, DC, United States; ^6^The University of Texas Health Science Center at Houston, Houston, TX, United States; ^7^Department of Psychiatry, Yale School of Medicine, New Haven, CT, United States

**Keywords:** family homelessness, homelessness prevention, rapid rehousing, SSVF program, veterans

## Abstract

Homelessness impacts entire families, with potential intergenerational consequences. Addressing family homelessness provides both immediate relief and long-term societal benefits. While various programs exist to mitigate homelessness, the United States (US) Department of Veterans Affairs’ (VA) Supportive Services for Veteran Families (SSVF) program offers a distinctive model for combating homelessness among veterans by supporting their families as well. We analyzed VA SSVF administrative data from 2014 to 2022, covering over 800,000 program entries from all SSVF beneficiaries in the US, to describe the sociodemographic profiles of SSVF veteran families—including children and adult family members of veterans. Families receiving SSVF assistance faced substantial economic and health-related challenges, including high unemployment, single-income dependency, and service-related disabilities. Children in these families represent a particularly vulnerable population, underscoring the need for targeted interventions to prevent long-term adverse outcomes. Our findings point to the role of SSVF in providing essential support for homeless veterans by also offering important services to their families. This broader approach offers lessons that may extend beyond the veteran community to address homelessness in individuals nationwide. Expanding coordinated, multi-agency approaches that build upon and modify the SSVF model could strengthen national efforts to reduce homelessness.

## Introduction

Approximately one-third of the United States (US) homeless population are families with children (1) who face immediate hardships and increased health risks that can extend throughout their lifetimes (2). While homelessness rates in the general US population have reached their highest level in recorded history, rates among some subpopulations, notably military veterans, have declined dramatically in recent years (3, 4). Understanding strategies that effectively support veterans and their families may offer insights for addressing similar challenges within the general population (5).

Supportive Services for Veteran Families (SSVF) is a US Department of Veterans Affairs (VA) program that has uniquely provided comprehensive services for rapid rehousing and homelessness prevention to veterans and their families, including children and non-veteran adult family members (NVAFM) (6). Funded by the nation’s largest healthcare system, SSVF distinguishes itself from other homeless assistance programs through its multi-faceted approach, addressing the complex interplay of economic, social, and other factors affecting housing stability by partnering with community-based organizations and other federal programs to provide homelessness prevention and rapid rehousing services for the entire family unit. These flexible, short- to medium-term components encompass robust services delivered in partnership and coordination with regional and local organizations within the communities where veterans live, while also connecting veteran families to other VA, federal, and community resources (7).

Through these federal-community partnerships, SSVF has consistently served veterans and veteran family members for over 10 years. Although previous studies have shown how SSVF supports individual veterans facing homelessness (8, 9), no prior work has focused on veteran family members, identified their sociodemographic characteristics, and documented the challenges they face. This brief report analyzes over 800,000 SSVF records (2014–2022) to document trends for children and NVAFM served, contextualized alongside trends in the general population, to highlight how lessons learned from SSVF might more broadly inform other programs serving homeless individuals in the US.

## Methods

We analyzed over 800,000 SSVF records (2014–2022) from the Homeless Management Information System (HMIS). The VA requires SSVF grantee organizations to collect some household-level data through HMIS, including sociodemographic characteristics of veterans receiving services from SSVF, which are aggregated by the VA National Center on Homelessness among Veterans in its centralized repository. The data for this study were retrieved from the repository for all entries and exits from 2012–2022. Data were separated by year of program entry. We then excluded data for the program’s first two years, 2012 and 2013, because the data structure and format for these initial years differed considerably from that of later years. We identified individuals who received services more than once in a calendar year, and included only their first service entry in our analysis. This approach allowed us to assess their characteristics and approximate annual descriptive statistics of the population served by SSVF each year. After restricting to participants with valid (e.g., non-missing) birthdate data and omitting erroneous cases, 533,671 records were connected to veterans, 166,119 to children, and 106,262 to NVAFM.

We focused on available HMIS and VA data aligned with the focus of this brief report, and conducted descriptive analyses to summarize the characteristics of individuals served by SSVF. For veterans, we analyzed age, generational cohort, gender, race/ethnicity, Iraq/Afghanistan (Operations Enduring Freedom/Iraqi Freedom/New Dawn) service record, employment at program entry, service-related disability, SSVF services, and household composition. For children and NVAFM, we examined age, gender, race/ethnicity, household composition, and SSVF services. Veteran characteristics were linked to child and NVAFM records via a family identifier variable. Although the dataset includes multiple years of data, we emphasize secular trends more selectively in cases where the pattern of results suggests that temporal variation could provide a meaningful interpretive lens.

## Results

While over 70% of veterans served by SSVF (2014–2022) lived in households of one person (veteran only), about one-third of veteran households included children (21%) and NVAFM (13%). SSVF families form various constellations, including veterans with (i) NVAFM only, (ii) children only, and (iii) NVAFM and children no longer living in the household (e.g., due to death or domestic violence) but still qualifying for SSVF services. These family forms contextualize trends in Table 1 (children) and 2 (NVAFM). Veteran data are reported in Supplementary Table 1, with additional statistics available in SSVF Annual Reports (https://www.va.gov/homeless/ssvf/research-library/).

**Table 1:** Sociodemographic characteristics of SSVF children from 2014–2022.

Characteristic	2014	2015	2016	2017	2018	2019	2020	2021	2022
Total (*N*)	26,797	26,427	22,031	20,319	17,218	15,646	14,264	10,379	13,038
Age, *M* (*SD*)	7.84 (5.02)	7.92 (5.02)	7.93 (5.01)	8.06 (5.00)	8.15 (4.96)	8.24 (4.95)	8.20 (5.00)	8.40 (5.01)	8.50 (4.96)
Female^a^, %	N/A	N/A	N/A	48.97	48.78	49.02	47.85	48.39	50.27
Racial or ethnic minority^b^, %	N/A	N/A	N/A	49.56	36.93	32.39	39.98	37.69	52.98
Single-adult household, %	30.57	29.44	31.21	31.30	33.92	34.53	28.83	31.69	31.58
Household with other minors, %	78.37	80.07	80.50	80.92	81.18	79.98	83.95	82.45	83.91
Household size, *M* (*SD*)	4.15 (1.59)	4.17 (1.62)	4.18 (1.64)	4.21 (1.67)	4.24 (1.69)	4.20 (1.70)	4.12 (1.64)	4.03 (1.67)	4.14 (1.68)
SSVF program, %									
Homelessness prevention	59.32	51.39	53.75	50.61	51.53	51.77	61.44	57.82	58.05
Rapid rehousing	40.68	48.61	46.25	49.39	48.47	48.23	38.56	42.18	41.95
Total (*N*) with veteran in household	25,831	25,564	21,128	19,421	16,405	13,978	13,519	9,750	12,615
OEF/OIF veteran, %	31.32	32.04	32.88	31.75	30.23	29.50	29.94	28.48	26.15
Female veteran, %	24.70	26.22	26.31	28.29	30.28	33.06	33.06	34.28	33.41
Racial or ethnic minority veteran^c^, %	48.77	50.58	52.59	46.33	37.05	34.74	40.32	44.79	52.21
Veteran not employed at entry, %	N/A	N/A	N/A	99.88	69.78	69.39	73.56	73.53	70.50
Veteran had any service-related disability^d^, %	67.31	68.33	69.85	70.90	70.66	71.34	72.90	73.17	70.62
Veteran had max. (100%) service-related disability^d^, %	18.43	17.90	17.79	16.93	17.10	16.95	16.01	15.77	12.90

SSVF, Supportive Services for Veteran Families; *M*, mean; *SD*, standard deviation; OEF/OIF, Operation Enduring Freedom/Operation Iraqi Freedom.

^a^Gender proportions for minors are out of total from whom gender information was collected (*n* = 3,189–10,923).

^b^Race and ethnicity proportions for minors are out of total from whom race and ethnicity information were collected (*n* = 3,139–17,125).

^c^Race and ethnicity proportions for veterans are out of total from whom race and ethnicity information were collected in a given year (*n* = 9,390–22,041).

^d^Employment proportions for veterans are out of total from whom employment information was collected (*n* = 2,602–13,910). The first year of employment data available for children (i.e., for the veterans linked to child cases) in 2017 was a very small subsample (2,602 out of 19,421 records), and therefore, it is likely not accurate due to this data issue.

^e^Service-related disability proportions for veterans are out of total from whom service-related disability information was collected (*n =* 8,231–22,949).

Table 1 shows the sociodemographic characteristics of children who received SSVF services. Most children were school-aged (around 8 years old), lived with other children and more than one adult, received homelessness prevention services, and were linked to unemployed veterans and those with service-related disabilities. Children enrolled in SSVF living in households with other children increased over the observation period. More than half of children received homelessness prevention services over the observation period, compared to about one-third of veterans (Supplementary Table 1). Over 40% of children enrolled in SSVF were actively homeless and received rapid rehousing assistance. About 3 in 10 children had a female veteran parent, compared to just over 1 in 10 veterans receiving SSVF services. The proportion of children connected to SSVF through a female veteran steadily increased over the study period. Around 70% of children had a veteran parent with a service-related disability, with small secular increases over the observation period, compared to about half of all veterans receiving SSVF services. Veterans with children had relatively lower unemployment rates, with 70–74% of children having an unemployed veteran parent, compared to 80–85% of all veterans enrolled in SSVF.

Table 2 describes the sociodemographic characteristics of NVAFM who received SSVF services. Most NVAFM were in their late 30s or early 40s, female, white, living in households with other people, and linked to unemployed veterans and those with service-related disabilities. Compared to veterans (Supplementary Table 1), more NVAFM lived in households with children (35–54% of NVAFM vs. 11–20% of veterans over the observation period). Similar to children, a higher proportion of NVAFM received homelessness prevention support compared to veterans enrolled in SSVF. While 46–54% of veterans had service-related disabilities, 57–60% of NVAFM lived in households with veterans who had disabilities.

**Table 2:** Sociodemographic characteristics of SSVF non-veteran adult family members from 2014–2022.

Characteristic	2014	2015	2016	2017	2018	2019	2020	2021	2022
Total (*N*)	17,070	16,425	13,351	12,160	10,726	9,889	10,453	7,369	8,819
Age, *M* (*SD*)	37.44 (13.75)	37.73 (13.90)	37.96 (14.00)	38.27 (14.00)	38.90 (14.46)	39.12 (14.67)	39.79 (14.82)	40.22 (15.06)	40.32 (15.41)
Female^a^, %	N/A	N/A	N/A	82.87	81.01	80.19	78.46	78.68	79.02
Racial or ethnic minority^b^, %	N/A	N/A	N/A	23.96	28.31	34.89	25.59	32.43	40.97
Household with children, %	51.52	49.64	48.97	48.62	45.35	44.48	40.29	38.00	40.03
Household size, *M* (*SD*)	3.29 (1.51)	3.24 (1.53)	3.24 (1.54)	3.27 (1.58)	3.19 (1.58)	3.19 (1.59)	3.06 (1.52)	2.99 (1.51)	3.07 (1.55)
SSVF program, %									
Homelessness prevention	57.18	48.46	50.18	46.25	44.70	45.83	52.77	50.10	51.63
Rapid rehousing	42.82	51.54	49.82	53.75	55.30	54.17	47.23	49.90	48.37
Total (*N*) with veteran in household	16,245	15,555	12,633	11,490	10,065	8,719	9,665	6,748	8,328
OEF/OIF veteran, %	21.19	21.43	21.66	20.24	18.02	16.68	16.17	15.15	15.03
Female veteran, %	11.28	11.37	10.82	12.80	13.50	13.96	14.27	13.90	14.04
Racial or ethnic minority veteran^c^, %	44.55	42.62	42.56	35.61	34.60	36.14	26.21	38.31	42.26
Veteran not employed at entry^d^, %	N/A	N/A	N/A	76.69	78.30	79.15	81.83	83.05	80.23
Veteran had any service-related disability^e^, %	59.99	58.90	59.88	60.39	58.98	58.23	58.10	57.78	57.15
Veteran had max. (100%) service-related disability^e^, %	14.73	13.97	13.70	13.32	12.52	11.13	11.15	10.66	9.56

SSVF, Supportive Services for Veteran Families; *M*, mean; *SD*, standard deviation; OEF/OIF, Operation Enduring Freedom/Operation Iraqi Freedom.

^a^Gender proportions for non-veteran others are out of total from whom gender information was collected (*n =* 1935–6,508).

^b^Race and ethnicity proportions for non-veteran adult family members are out of total from whom race and ethnicity information were collected (*n =* 1904–10,631).

^c^Race and ethnicity proportions for veterans are out of total from whom race and ethnicity information were collected (*n =* 6,481–13,865).

^d^Employment proportions for veterans are out of total from whom employment information was collected (*n =* 3,351–8,508).

^e^Service-related disability proportions for veterans are out of total from whom service-related disability information was collected (*n =* 5,556–14,003).

## Discussion

SSVF distinctively recognizes that homelessness affects not just individual veterans but also their families. Since 2012, SSVF has supported over 800,000 veterans and their family members, working through community grantees and other federal agencies to offer a wide range of services and supports. Such efforts include temporary financial assistance for rent and other needs, healthcare access and navigation, family reunification, job training programs, and employment resources. Alongside other VA programs, SSVF has helped reduce veteran homelessness, despite rising national trends in the general population. Our analysis documents the sociodemographic characteristics of veteran families as they confront major challenges that also impact the general population (10).

While more than 70% of SSVF veterans lived in single-person households, about one-third of individuals served by SSVF were children and NVAFM. Compared to single SSVF veterans, many children and NVAFM lived in a household with a heightened need for resources and support. Over half of SSVF children and NVAFM lived with veterans with service-related disabilities, limiting employment opportunities and increasing social, economic, and health challenges for the household. Two-thirds of children lived with unemployed veterans (although unemployment rates for veterans with children were lower than for veterans without children); a factor that can heighten the risk of homelessness. Half of children received homelessness prevention services (i.e., rent assistance to stay housed), meaning these children were on the brink of homelessness when they received assistance, compared to about one-third of veterans. This suggests that children are able to access housing support and other services through SSVF *before* losing their housing, which helps mitigate the risk of homelessness and its adverse health and economic consequences. Over 40% of children enrolled in SSVF were already homeless and received rapid rehousing assistance, demonstrating that SSVF serves as a vital lifeline by providing support to help children and their families quickly find more stable housing.

Most SSVF children were school-aged, a segment of the general population experiencing increasing homelessness rates (11). SSVF services increasingly reached households with more than one child, represented by the small increases over time in the proportion of children enrolled in SSVF who lived with other children. Similar to the general homeless population, about one-third of SSVF children lived in single-parent households. These households are often headed by female veterans, and the proportion of children receiving SSVF services through female veterans increased over the observation period. Households headed by single parents often face financial strains (12), and while SSVF offers flexible supplementary income for expenses like childcare, working parents may still struggle to find suitable childcare and flexible employment. SSVF housing services and financial assistance mitigate the immediate effects of homelessness and promote long-term stability, but additional efforts are needed to support working parents’ employment within the VA and the general population.

Our findings also resonate with prior research suggesting the need for a multi-pronged strategy to address the complex needs of homeless families. Aware of the link between childhood homelessness and higher risks of adverse experiences that can carry long-term health implications (13, 14), SSVF coordinates with grantees and VA Medical Centers to provide veterans with treatment that mitigates homelessness risk factors (e.g., substance use, mental disorders) and connects children to non-VA services. SSVF encourages veterans and their families to engage social support networks (e.g., friends and family) and offers financial assistance to family and friends willing to rehouse them, recognizing that strategic and coordinated partnerships, not just single organizations, are essential to providing comprehensive care for the individual and the whole family.

This descriptive analysis of families served by the SSVF program highlights the complex and intersecting challenges that many veteran households face, including housing insecurity, unemployment, disability, and childcare and healthcare needs. The SSVF model addresses these needs by connecting families to a wide range of community-based services and resources. This integrated approach could inform homelessness programs beyond the veteran population. Expanding coordinated, multi-agency efforts that coordinate with local community-based organizations based on the SSVF model could strengthen national efforts to reduce homelessness by addressing both individual and family needs, including childcare, social support, healthcare access, and employment and disability issues.

### Limitations and future directions

While the present essay documents, for the first time, the characteristics of children and adult family members of veterans who received services through SSVF over a long (8-year) evaluation period, the breadth, depth, and temporal coverage of some variables in our analysis were limited by the availability of data. Inconsistent reporting of housing status at program entry, particularly in the early years of SSVF, limited trend comparisons. Although our analysis assessed trends over time in veterans’ service-related disabilities, an important indicator of health, we lacked data on specific health conditions underlying veterans’ service-related disabilities. Future research should examine categories of disabilities to assess differential associations with important outcomes (e.g., employment). While SSVF’s data infrastructure serves administrative needs, improving data quality and linking it to other sources (e.g., prospective longitudinal datasets with comprehensive health and employment assessments) could enhance program effectiveness and broader homelessness reduction efforts. Additional work in this area could employ random sampling to identify SSVF service recipients for in-depth qualitative interviews, which might help to contextualize barriers and resource gaps not fully captured by quantitative data. Future studies could also explore longitudinal outcomes for children and NVAFM receiving SSVF services.

## Conclusion

Our findings highlight the complex needs of homeless and at-risk veteran families served by SSVF, particularly the difficult and distinct needs of children and non-veteran adult family members (15). Since its inception in 2012, SSVF has not only prioritized supporting housing stability but also contributed to addressing broader social, economic, and health needs of veterans and their families by providing both immediate assistance to those experiencing homelessness and preventive support to those at risk (6). As one of the largest family homelessness programs in the US, the multi-faceted and coordinated approach of SSVF offers an example of coordinated care for the complex needs of families and may provide a useful example for other organizations serving homeless families. Scaling and sustaining such comprehensive programs could transform the public health response and help efforts to make homelessness rare, brief, and non-recurring for all populations at risk.

## Data Availability

The data analyzed in this study is subject to the following licenses/restrictions: de-identified data were obtained from the VA NCHAV. VA has ethical guidelines for data security and confidentiality and data access may be obtained through an application and credentialing process. Requests to access these datasets should be directed to Jack Tsai, jack.tsai@uth.tmc.edu.
