# Supplementary material for: Families served during the first decade of the Supportive Services for Veteran Families program: a descriptive analysis
Source: Front Public Health. 2025 Jul 24;13:1634259. doi: 10.3389/fpubh.2025.1634259 (PMC12328279; doi:10.3389/fpubh.2025.1634259)
Supplement: Supplementary file 1 [file Table_1.pdf]

Supplementary Table 1. Sociodemographic characteristics of SSVF veterans from 2014-2022.

Characteristic	2014	2015	2016	2017	2018	2019	2020	2021	2022
Total (*N*)	73358	75516	64330	60574	55143	52638	59158	44026	48928
Age, *M* (*SD*)	48.37 (12.71)	48.92 (12.78)	49.36 (13.08)	49.77 (13.23)	50.61 (13.31)	51.08 (13.43)	51.95 (13.53)	52.27 (13.97)	52.31 (14.32)
Generational cohort, %									
Silent Generation (born 1928-45)	3.57	3.26	3.44	3.24	3.14	2.84	2.75	2.75	2.57
Baby Boomer Generation (born 1946-64)	52.67	52.06	50.05	48.39	48.53	47.37	47.24	45.80	43.30
Generation X (born 1965-80)	26.56	26.64	26.74	27.37	27.29	27.60	27.73	26.93	27.41
Millennial Generation (born 1981-96)	17.20	18.04	19.72	20.90	20.72	21.70	21.42	23.00	24.26
Generation Z (born 1997-2012)	0.00	0.01	0.05	0.10	0.33	0.50	0.87	1.53	2.46
Served in OEF/OIF, %	15.22	15.07	14.91	14.39	13.24	12.54	11.74	11.70	11.34
Female, %	13.25	12.83	12.48	13.31	13.58	13.59	12.80	13.21	13.73
Racial or ethnic minority[a], %	49.70	48.19	46.94	43.91	39.70	39.71	39.57	40.53	44.88
Not employed at program entry[b], %	N/A	N/A	N/A	80.11	81.10	81.94	85.81	85.84	83.81
Any service-related disability[c], %	54.26	53.18	46.07	54.25	53.68	53.10	51.70[r]	51.90	52.43
Max. (100%) service-related disability[c], %	13.23	12.07	10.13	9.99	11.63	10.29	9.45[r]	8.62	7.81
Single-person household, %	71.49	73.18	73.77	74.69	76.27	77.08	79.19	79.82	78.32
Household with children, %	18.36	17.07	16.64	15.96	14.60	13.91	11.53	11.30	12.44
Household size, *M* (*SD*)	1.60 (1.18)	1.56 (1.16)	1.55 (1.14)	1.53 (1.14)	1.49 (1.10)	1.47 (1.07)	1.41 (0.99)	1.38 (0.95)	1.42 (1.01)
SSVF Program, %									
Homelessness Prevention	39.14	30.99	31.87	29.30	28.10	28.57	35.60	35.32	37.11
Rapid Rehousing	60.86	69.01	68.13	70.70	71.90	71.43	64.40	64.68	62.89

Note. SSVF = Supportive Services for Veteran Families, *M* = mean, *SD* = standard deviation, OEF/OIF = Operation Enduring Freedom/Operation Iraqi Freedom.

[a]race and ethnicity proportions are out of total from whom race and ethnicity information were collected (*n*=42272-66222).

[b]employment proportions are out of total from whom valid employment information was collected (*n*=16806-57477).

[e]service-related disability proportions are out of total from whom service-related disability information was collected (*n*=31914-65048).
